# Cl*RTL1* Encodes a Chinese Fir RNase III–Like Protein Involved in Regulating Shoot Branching

**DOI:** 10.3390/ijms161025691

**Published:** 2015-10-26

**Authors:** Xia Li, Qian Su, Renhua Zheng, Guangxin Liu, Ye Lu, Liming Bian, Jinhui Chen, Jisen Shi

**Affiliations:** 1 Key Laboratory of Forest Genetics and Biotechnology of the Ministry of Education of China, Nanjing Forestry University, Nanjing 210037, China; E-Mails: lixia1069@ujs.edu.cn (X.L.); suqian@njfu.com.cn (Q.S.); guangxinliu@njfu.com.cn (G.L.); luye_njfu@126.com (Y.L.); lmbian@njfu.edu.cn (L.B.); 2 School of the Environment and Safety Engineering, Jiangsu University, Zhenjiang 212013, China; 3 Southern Mountain Timber Forest Cultivation Lab, Fujian Academy of Forestry, Ministry of Forestry, Fuzhou 350012, China; E-Mail: zrh08@126.com

**Keywords:** *Cunninghamia lanceolata* (Lamb) Hook, branching, cDNA amplified fragment length polymorphism analysis, real time-PCR, overexpression, axillary bud

## Abstract

Identification of genes controlling shoot branching is crucial for improving plant architecture and increasing crop yield or biomass. A branching mutant of Chinese fir named “Dugansha” (*Cunninghamia lanceolata* var. *dugan.*) has been isolated in our laboratory. We chose the cDNA-AFLP technique and an effective strategy to screen genes that potentially regulate shoot branching in Chinese fir using this mutant. An RNase III-like1 cDNA fragment named Cl*RTL1* was identified as a potential positive regulator. To investigate the function of Cl*RTL1* in regulating shoot branching, we cloned the full-length cDNA sequence from *C. lanceolata* (Lamb.) Hook, deduced its secondary structure and function, and overexpressed the coding sequence in *Arabidopsis*. The Cl*RTL1* cDNA is 1045 bp and comprises an open reading frame of 705 bp. It encodes a protein of 235 amino acids. The deduced secondary structure of the ClRTL1 indicates that it is a mini-RNase III-like protein. The expression analysis and phenotypes of 35S: Cl*RTL1* in *A. thaliana* implies that Cl*RTL1* plays a role in promoting shoot branching in Chinese fir.

## 1. Introduction

Shoot branching is important for the establishment of plant architecture and is a key factor in plant yield (crops) or biomass allocation (trees). Thus, studies on the mechanism of shoot branching have scientific and economic importance. The shooting process generally involves two developmental stages, as follows: initiation of axillary meristems in the leaf axils; and the outgrowth of axillary buds. The fates and activities of axillary meristems are regulated by genetic programs, and environmental stimuli channeled through interacting hormonal and transcription factor regulatory networks [1]. The Beveridge group recently revealed that axillary bud outgrowth was inhibited by the strong sugars flowing convergence to the shoot tips, which gives us a new perspective to overturn the long-standing hypothesis on apical dominance [2].

Identification of genes controlling shoot branching is crucial because these genes are key targets that can be manipulated to improve plant architecture and increase crop yield or biomass [3]. Several genes regulating shoot branching have been identified in various species, such as *Arabidopsis*, tomato, rice, tobacco, maize, pea, and petunia. The GRAS family of transcription factors includes members that are critical switches in the development of axillary meristems **(**AMs) such as *LATERAL SUPPRESSOR* (*LS*) in tomato, *LATERAL SUPPRESSOR* in *Arabidopsis* (*LAS*), and its rice ortholog *MONOCULM1* (*MOC1*) [4,5,6]. *TOMATO BLIND* (*BL*), the *Arabidopsis REGULATOR OF AXILLARY MERISTEMS* (*RAX*), *LAX PANICLE* (*LAX*), and *SMALL PANICLE* (*SPA*) control the formation of lateral meristems by encoding MYB (MYB repeat domain) or bHLH (a basic helix-loop-helix domain) transcription factors [7,8,9]. Maize *TEOSINTE BRANCHED1* (*TB1*), the rice ortholog of *TB1* (Os*TB1*), *Arabidopsis BRANCHED1* (*BRC1*), and pea *BRANCHED* (*BRC*) may suppress the growth of axillary meristems [10,11,12]. *Arabidopsis SUPERSHOOT/BUSHY* (*SPS/BUS*), which encodes a protein of the cytochrome P450 superfamily, alters shoot branching by modulating cytokinin or auxin level [13,14]. Overexpression of petunia *LATERAL shoot INDUCING FACTOR* (*LIF*) in petunia, tobacco, or *Arabidopsis* dramatically decreases lateral shoots, indicating its conserved role in defining plant architecture [15]. A subset of orthologous genes including *MORE AXILLARY BRANCHING1–4* (*MAX1–4*) in *Arabidopsis*, *RAMOSUS1–5* (*RMS*1–5) in pea, *DECREASED APICAL DOMINANCE1–3* (*DAD1–3*) in petunia hybrids, *DWARF* (*D*), and *HIGH TILLERING DWARF* (*HTD*) in rice down-regulate shoot branching by functioning in the biosynthesis or signaling pathway of strigolactone [16]. Although great progress has been achieved in genetic control of shoot branching, the mechanisms involved are still not fully understood, especially in trees.

**Figure 1:**
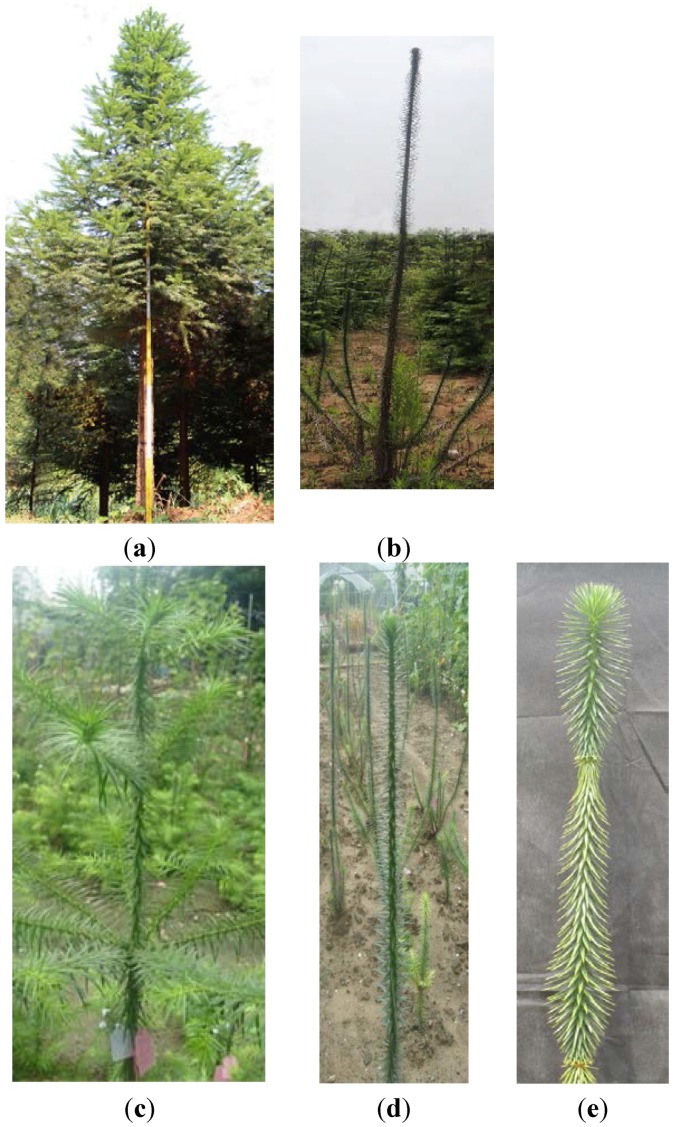
Phenotypes of the Chinese fir mutant line “Dugansha” and the respective wild-type No. 020. (**a**) Six-year-old tree of the Chinese fir elite genotype No. 020 (wild type), H (Height) 10.5 m, DBH (Diameter at breast height) 12 cm; (**b**) The Chinese fir mutant “Dugansha” (6 years old), H 3.0 m, DBH 2 cm; (**c**) Two-year-old cutting of the Chinese fir elite genotype No. 020 (wild type); (**d**) Two-year-old cutting of the Chinese fir mutant “Dugansha”; (**e**) Annual height growth (between the white dash-arrow bar) of “Dugansha”.

In our laboratory, a branching mutant of Chinese fir *Cunninghamia lanceolata* (Lamb.) Hook named “Dugansha” (*C. lanceolata* var*.* “Monocaulis” Yieh*.*) was identified in 1973 and has since then been reserved in the clonal archives [17]. It can only be vegetatively propagated and it has never flowered during the last 42 years. The top settled bud (main bud) of the mutant keeps sprouting out each spring. Therefore, the main stem can grow continuously in height, but with no branch and no diameter increment. The mutant might reach 8–10 m in height or higher if the mechanical support is properly supplied. If not, the single stem mutant “Dugansha” (“Dugansha” in Mandarin Chinese means “single stem”) might bend over or fall down (Figure 1).

A comparative gene expression study was performed between wild-type Chinese fir No. 020 and its shoot branching mutant “Dugansha” using cDNA-amplified fragment length polymorphism (cDNA-AFLP) to identify genes that modulate shoot branching. *C. lanceolata* RNase III-like gene 1 (Cl*RTL1*) was identified as one of the differentially expressed cDNA fragments homologous to RNase III of *Escherichia coli*.

**Figure 2:**
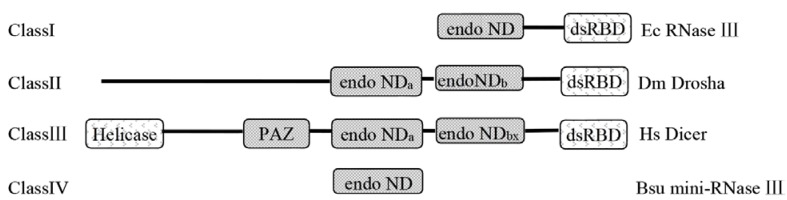
Schematic structures of class I–IV RNase III proteins. Ec RNase III: *E. coli* RNase III, SWISS-PROT PROT P05797; Dm Drosha: *D. melanogaster* Drosha, SWISS-PROT Q9XYN5; Hs Dicer: *H. sapiens* Dicer, GenBank AB028449; Bsu mini-RNase III: *B. subtilis* mini-RNase III (SWISS-PRO C69742). Boxes with black back ground represent catalytic endonuclease domains (endo ND) and PAZ domains (PAZ); Boxes with white back ground represent dsRNA-binding domains (dsRBD) and N-terminal DEXH-box RNA helicase domains (Helicase).

Ribonuclease III (RNase III) belongs to the family of dsRNA-specific endoribonucleases characterized by the presence of a highly conserved nine amino acids in the catalytic center known as the RNase III signature motif (_37_ETLEFLGDA_45_ in *Aquifex aeolicus* RNase III), which is found in all studied prokaryote and eukaryote RNase IIIs [18,19,20,21,22,23]. Four structural classes of RNase III molecules have been described so far (Figure 2). The first class is the simplest RNase III protein, found in bacteria, yeast, and plant. This protein consists of an N-terminal endonuclease domain (endo ND) and a C-terminal double-stranded RNA binding domain (dsRBD). The second class comprises *Drosophila melanogaster* Drosha protein and its homologs, which have two endoNDs, one dsRBD, and a large N-terminal extension [18]. The third class of RNase IIIs were presented by *Homo sapiens* RNase III (Hs-Dicer) that consists of two endoNDs, one dsRBD, and an even larger N-terminal extension, which includes a helicase domain and a PAZ domain [24]. Class 4 RNase IIIs, represented by *Bacillus subtilis* Mini-III having just one catalytic domain [25].

In the current study, to investigate the function of Cl*RTL1* in regulating shoot branching, we cloned the full-length cDNA sequence, deduced its secondary structure and function, and overexpressed the coding sequence in *Arabidopsis*.

## 2. Results

### 2.1. Isolation and Identification of Shoot Branching Genes Using the cDNA-AFLP Technique

**Figure 3:**
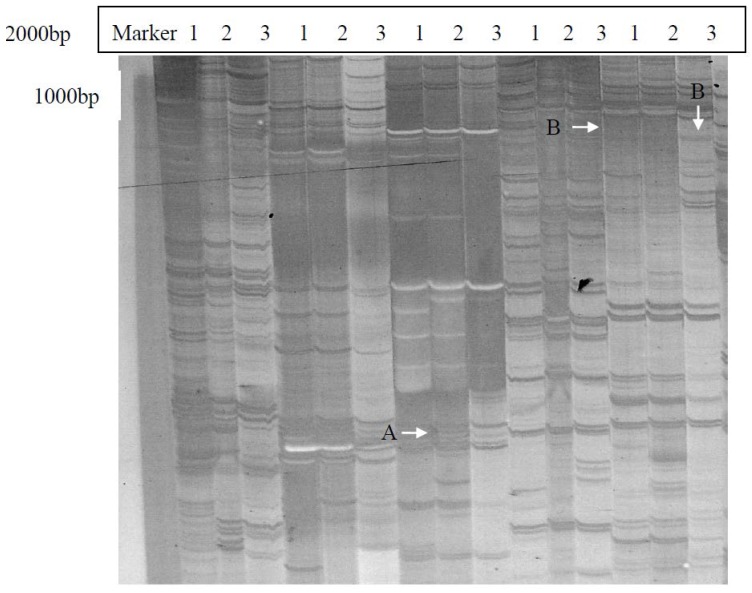
Differentially expressed cDNA fragments on a silver-stained cDNA-AFLP PAGE gel. (1) Primary SAMs of Chinese fir No. 020 that start elongation (are initiating AMs). The materials were obtained on 6 May; (2) Primary SAMs of Chinese fir No. 020 in un-elongation (before AMs initiation). The materials were obtained on 15 April 2009; (3) Primary SAMs of Chinese fir mutant “Dugansha”. The materials were obtained on 6 May 2009. Fragment “A”: Differentially expressed cDNA fragment that potentially regulate shoot branching positively Fragment “B”: Differentially expressed cDNA fragments that potentially regulate shoot branching negatively.

The cDNA-AFLP technique was applied to isolate genes involved in shoot branching of Chinese fir. A combination of *Mse* I and *EcoR* I restriction enzymes and 256 primer pairs were utilized in the screening of differentially expressed genes. To ensure stability and efficiency of the differential display, we established and optimized the cDNA-AFLP reaction system for Chinese fir No. 020 [26]. We defined the SAMs from the topical bud of the main stem as the “Primary SAMs,” and the SAMs from the top of the branches as the “Secondary SAMs.” “Primary SAMs” from elongated and not-yet-elongated primary SAMs of Chinese fir No .020 were collected on 6 May and 15 April. The “Primary SAMs” from “Dugansha” were obtained on 6 May (Figure 3). cDNA fragments expressed in Chinese fir No. 020 primary SAMs that start elongation were identified as potential positively regulating genes (Figure 3A). The cDNA fragments expressed both in the sample from the primary SAMs of branchless mutant “Dugansha” and from the wild type primary SAMs sample in un-elongation were identified as potential negatively regulating genes (Figure 3B). Following our screening strategy, 65 cDNA fragments were identified as potential positive or negative regulators of branching (Figure 3) based on their differential expression between these three types of primary SAMs analyzed. Cl*RTL1* was identified as a potential positive regulator of shoot branching expressed only in primary SAMs of Chinese fir No. 020 that are to start elongation (Figure 3; Fragment A). Since the homolog of Cl*RTL1* in *Arabidopsis*, Dicer-liker1 (DCL1), has also been reported to affect shoot branching [21,27], we focused on further investigating the function of this gene in Chinese fir.

**Figure 4:**
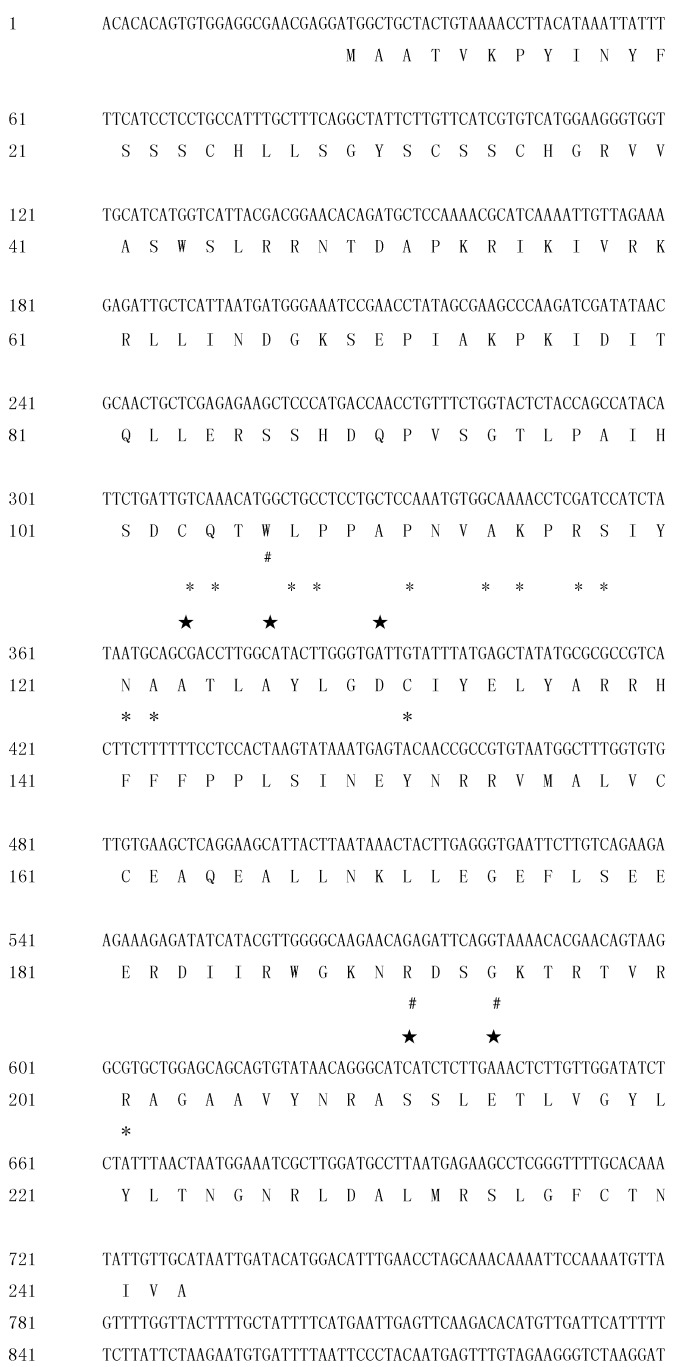
Full length cDNA sequence and deduced protein sequence of Cl*RTL1*. ★: active site; #: metal binding site; *: dimerization interface.

### 2.2. Cloning and Sequence Analysis of the Full-Length ClRTL1 cDNA Sequence

The full-length cDNA sequence of Cl*RTL1* was obtained by Rapid Amplification of cDNA Ends (RACE)-PCR. The corresponding full-length cDNA (1045 bp) was amplified with specific primers. The sequencing of the full-length cDNA showed that its coding sequences are 100% identical to the full-length cDNA sequence of Cl*RTL1*, which was obtained by RACE. Sequence analysis revealed that the Cl*RTL1* cDNA sequence contains a 5ʹ-untranslational region (UTR) of 25 bp, an open reading frame of 705 bp that encodes 235 amino acids and a 3ʹ-UTR of 312 bp (Figure 4). It encodes a protein of 235 amino acids with an estimated molecular mass of 28.9 kDa. NCBI Blastp indicated that the deduced amino acid sequence of this gene is highly homologous (69% identical) with RNase III-like protein of *A. thaliana* (9 × 10 ^−61^). The SMART tool and the Conserved domain database (CCD) search by PSI-BLAST indicates that the gene encodes a Ribonuclease III family protein with five active sites, three metal binding sites, and 13 dimerization interfaces (Figure 4). The full-length cDNA sequence of Cl*RTL1* was submitted to GenBank database (Accession No: KM587888).

### 2.3. The Deduced Secondary Structure of ClRTL1

**Figure 5:**
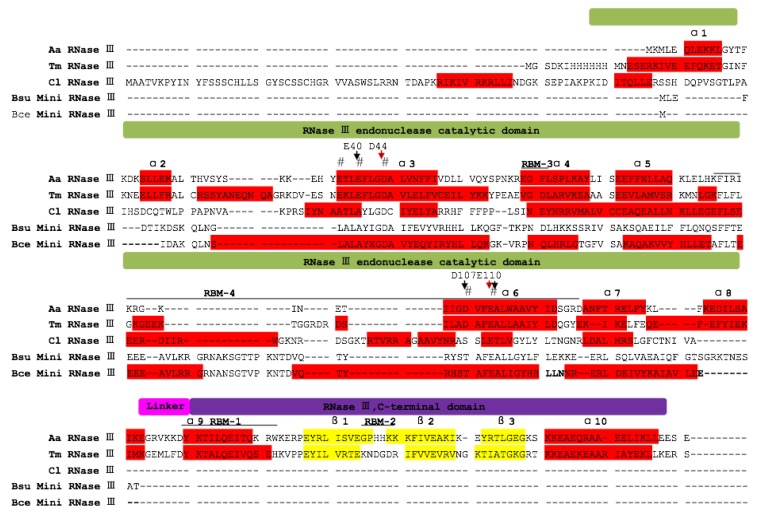
Alignment of the deduced amino acid sequence of Cl*RTL1* with four bacterial RNase III proteins. Amino acids corresponding to *A. aeolicus* (Aa) RNase III (PROTEIN DATA BANK 2NUE), *T. maritima* (Tm) RNase III (PROTEIN DATA BANK 10OW), *B. cereus* (Bce) mini-RNase III (PROTEIN DATA BANK 1U61), and *B. subtilis* (Bsu) mini-RNase III (SWISS-PRO C69742) are aligned with Cl*RTL1*. The secondary structures are indicated with shading, as follows: α1–α10, α-helix are shaded in red; and β1–β3, β-sheet are shaded in yellow; the catalytic, dsRNA binding, and linker domains are depicted above the sequence alignment in green, purple, and pink, respectively; the positions of RBM1–4 are shown. Catalytic residues are indicated by #. The key catalytic residues, D44 and E110 (*A. aeolicus* numbering), are shown with red arrows; E40, D107, and E110, which play roles in Mg^2+^ binding to RNase III, are shown with black arrows.

The second structure and alignment of the deduced ClRNase III–Like Protein 1 (ClRTL1) amino acid sequence with *A. aeolicus* RNase III (PROTEIN DATA BANK 2NUE), *Thermotoga maritima* RNase III (PROTEIN DATA BANK 10OW), *B. cereus* mini-RNase III (PROTEIN DATA BANK 1U61), and *B. subtilis* mini-RNase III (SWISS-PRO C69742) showed that the ClRTL1 only contained the endonuclease domain without the dsRNA binding domain and the linker domain between them (Figure 5). ClRTL1 has only one (RBM3) of the four RNA binding motifs with an extra α5b helix instead of the RBM4 (Figure 5).

The conserved signature sequence of RNase III family ATLAYLGDC (_37_ETLEFLGDA_45_ in *A. aeolicus* RNase III) was identified in ClRTL1 (Figure 5), thereby suggesting that this protein is a member of the RNase III family. The key amino acids Lys and Asn (K86 and N87, *A. aeolicus* mini III numbering), which were proposed to be in contact with the RNA helix [25], were found conserved at the loops between α5b and α6 in ClRTL1 (Figure 5). Residues equivalent to the Aa-RNase III D44 and E110 which are essential to the dsRNA processing center [28] were conserved in the ClRTL1 (Figure 5). E40 and D107 (*A. aeolicus* numbering), which play a role in Mg^2+^ binding to RNase III [29,30,31,32] are absent from ClRTL1 as in Bsu mini-RNase III protein.

**Figure 6:**
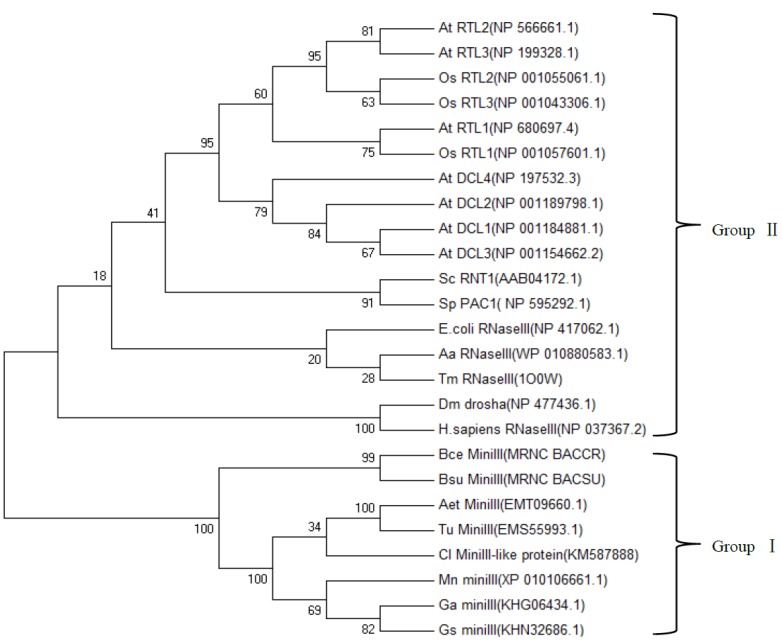
Phylogenetic tree of Cl*RTL1* deduced protein with other numbers of the RNase III family. The phylogenetic tree was constructed using MEGA 6.0 with the Neighbor-Joining method, following CLUSTAL X. The number at each node presents the percentage of bootstrapping after 1000 replications. At: *A. thaliana*; Os: *O. sativa*; Sc: *Saccharomyces cerevisiae*; Sp: *Schizosaccharomyces pombe*; E.coli: *E. coli*; Aa: *A. aeolicus*; Tm: *T. Maritima*; Dm: *D. melanogaster*; H. sapiens: *H. sapiens*; Bce: *B. cereus*; Bsu: *B. subtilis*; Aet: *A. tauschii*; Tu; *T. urartu*; Cl: *C. lanceolata*; Mn: *M. notabilis*; Ga: *G. arboreum*; Gs: *G. soja*. Cl*RTL1* deduced protein was highlighted with a black box.

### 2.4. Phylogenetic Analysis of ClRTL1 and Its Homologous Proteins

The RNase III phylogenetic tree is composed of the following two major groups (Figure 6). Group I contains only the mini-RNase III from different organisms, including *B. cereus*, *B. subtilis*, *Aegilops tauschii* (goatgrass)*, Triticum urartu* (common wheat), *C. lanceolata* (Chinese fir), *Morus notabilis* (mulberry), *Gossypium arboretum* (cotton), and *Glycine soja* (soybean). Group II comprises three other classes of RNase IIIs, such as *E. coli* RNase III, *D. melanogaster* Drosha, *H. sapiens* Dicer, *A. aeolicus* Dicer-like protein (DCL), and *Oryza sativa* Dicer-like protein (DCL). The ClRTL1 was clustered into the group of mini-RNase III. The relationship displayed in the phylogenic tree was generally in agreement with the traditional taxonomy (Figure 6).

### 2.5. Relative Expression Analysis of *Cl*RTL1

**Figure 7:**
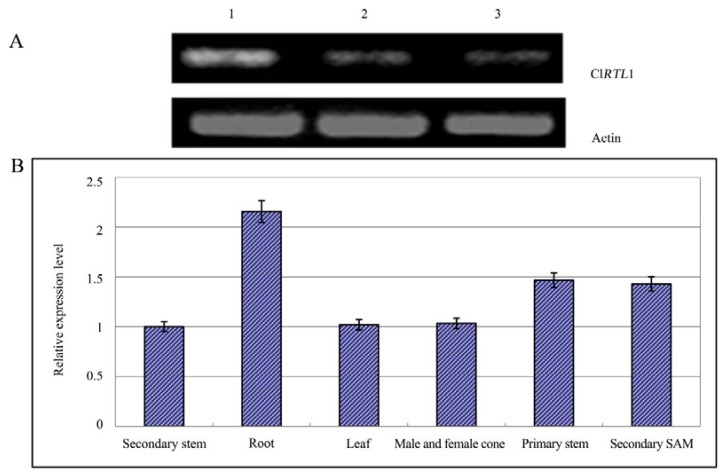
Expression analysis of Cl*RTL1*. (**A**) Semi-quantitative expression analysis of Cl*RTL1* in three types of SAMs. (1) Primary SAMs of Chinese fir No. 020 that are initiating AMs. The materials were obtained on 6 May; (2) Primary SAMs of Chinese fir No. 020 before AMs initiation. The materials were obtained on 15 April; (3) Primary SAMs of Chinese fir mutant “Dugansha”. The materials were obtained on 6 May; (**B**) Expression analysis of Cl*RTL1* in different tissues of Chinese fir. Real-time PCR was conducted using the cDNA of two-year-old Chinese fir as the template. Actin was used for normalization. The error bars indicate the standard deviation. Results were the mean of at least three biological replicates.

As shown in Figure 7A, primary SAMs of wild type plants that have started initiating AMs displayed higher Cl*RTL1* transcription levels than wild type samples before AM initiation and in the respective tissue of “Dugansha”. This result further supported the role of Cl*RTL1* in Chinese fir shoot branching and highlighted the high isolation efficiency of our screening strategy through cDNA-AFLP (Figure 7A). Expression analysis in different plant tissues by real-time PCR showed that while the gene was expressed in all Chinese fir tissues tested, the levels of expression were high in the root, intermediate in the primary stem and the secondary SAM, and low in male and female cones, leaf, and secondary stems (Figure 7B).

**Figure 8:**
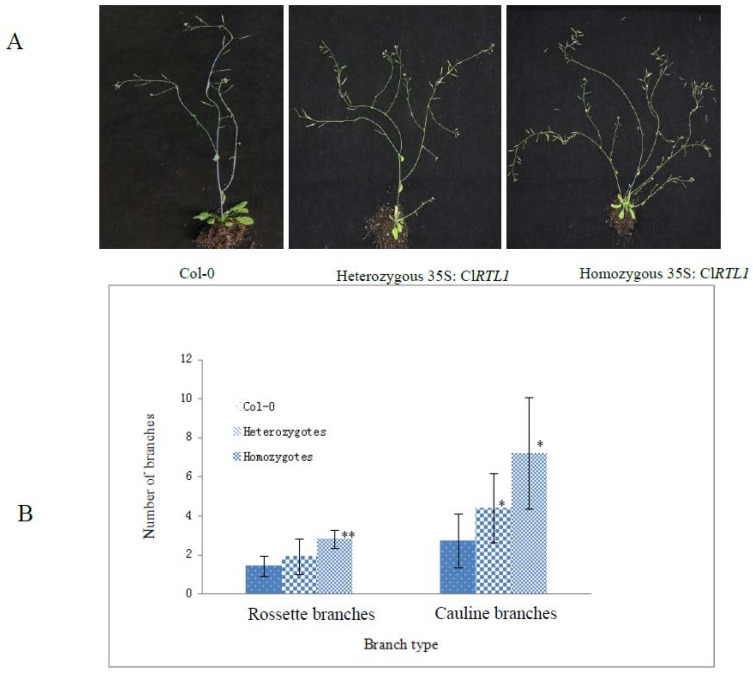
The increased shoot branching phenotypes of 35S*:* Cl*RTL1* seedlings. (**A**) 35S*:* Cl*RTL1* seedlings showing increased shoot branching (at four weeks after transplanting) Col-0: wild type *A. thaliana* (ecotype Columbia). Heterozygous 35S*:* Cl*RTL1* seedlings which showed a higher number of cauline branches than wild-type plants. Homozygous 35S*:* Cl*RTL1* seedlings which showed more severe phenotype with significantly higher numbers of cauline branches and rosette branches than wild-type plants; (**B**) Quantitative analysis of the secondary shoot development in 35S: Cl*RTL1* seedlings. Rosette branches and cauline branches were counted at four weeks after transplanting. Error bars represent the standard errors of the means: *n* = 10–20. * *p* < 0.05, ** *p* < 0.01. Col-0: wild-type *A. thaliana* (ecotype Columbia); Heterozygotes: Heterozygous 35S: Cl*RTL1 A. thaliana* seedlings; Homozygotes: Homozygous 35S: Cl*RTL1 A. thaliana* seedlings.

### 2.6. Phenotypes of CaMV 35S: ClRTL1 A. thaliana

To verify Cl*RTL1*’s functions in regulating shoot branching, the Cl*RTL1* cDNA sequence under the control of CaMV35S promoter was introduced into *A. thaliana*. The expression of Cl*RTL1* in transgenic *A. thaliana* was confirmed by amplifying the gene from leaf tissues by using PCR and RT-PCR (Figure S1). The 35S: Cl*RTL1* transgenic plants exhibited phenotypes of reduced apical dominance and vigorous shoot branching (Figure 8A). As shown in Figure 8B, the numbers of shoot branches of 35S: Cl*RTL1* transgenic plants significantly increased. Heterozygotes of 35S: Cl*RTL1* plants had significantly higher number of cauline branches than wild-type plants (Columbia-0). Homozygotes of 35S: Cl*RTL1* had a more server phenotype with significantly higher numbers of cauline branches and rosette branches than wild-type plants. The average number of cauline branches in the wild type *Arabidopsis*, heterozygous 35S: Cl*RTL1 Arabidopsis*, and homozygous 35S: Cl*RTL1 Arabidopsis* were 2.71 ± 1.38, 4.4 ± 1.79, and 7.2 ± 2.86, respectively. The average number of rosette branches in the wild *Arabidopsis*, heterozygous 35S: Cl*RTL1 Arabidopsis*, and homozygous 35S: Cl*RTL1 Arabidopsis* were 1.43 ± 0.53, 1.93 ± 0.91, and 2.8 ± 0.45, respectively.

## 3. Discussion

### 3.1. Effective Screening Strategy for ClRTL1 Identification

cDNA-AFLP technique is a reproducible, stable, and reliable method that is widely used to systematically analyze the transcriptomes of organisms or screen their differentially expressed genes [33,34,35]. In this research, we chose cDNA-AFLP technique to isolate genes potentially involved in the shoot branching of Chinese fir. We further developed an effective strategy for screening genes that regulate shoot branching using the branching mutant “Dugansha”. Differentially expressed genes between Chinese fir No. 020 primary SAMs that were about to start elongation and primary SAMs in un-elongation were preliminarily identified as potential positive or negative regulators of shoot branching. Simultaneously, the expression patterns of these genes in “Dugansha” were analyzed to confirm if these regulating genes played important roles in producing mutant phenotypes. This strategy greatly improved the efficiency of gene screening. Semi-quantitative reverse-transcription PCR (RT-PCR) and functional analysis results of Cl*RTL1* in *Arabidopsis* further indicated a role for Cl*RTL1* in Chinese fir shoot branching and verified the high isolation efficiency of the technique. Therefore, we suggest that cDNA-AFLP technique is a highly efficient technique to screen differently expressed genes with special strategy design.

### 3.2. ClRTL1 Regulates the Development of Chinese Fir by Promoting Shoot Branching

The present research revealed that Cl*RTL1* was a gene that played roles in shoot branching. Semi**-**quantitative expression analysis of Cl*RTL1* in three types of primary SAMs implied its role in shoot branching. The branching patterns and increased numbers of rosette branches and cauline branches of 35S: Cl*RTL1* plants further verified this hypothesis. Furthermore, the rosette leaves of Col-0 and 35S mutants showed no difference both in number and shape (Figure 8A and Table S1). Thus, we suggested that the increase in rosette branches of 35S: Cl*RTL1* seedlings was not due to increased phytomer number but to increased frequency of bud outgrowth. *DCL1*, the homologous gene of Cl*RTL1* in *Arabidopsis*, has been previously reported to affect shoot branching. Abnormal proliferation of shoot meristem cells and the loss of axillary meristems occurred in *DCL1* loss-of-function mutants [21,27]. Accordingly, Cl*RTL1* could promote shoot branching in Chinese fir by increasing the outgrowth of lateral buds.

Real-time PCR showed that the gene was detected throughout the plant. This result was consistent with *Arabidopsis* Dicer-like1 (*DCL1*) gene, whose transcript is present in the vegetative shoot apical meristem, inflorescence meristem, developing floral meristem, veins, central vasculature (especially in phloem cells), cotyledons, hypocotyl, and roots [21,36]. *DCL1* also reportedly has a broad regulatory function and plays essential roles in the growth and development of *Arabidopsis* [37]. *DCL2* and *DCL3* transcripts accumulated to detectable levels in inflorescence tissues, but not in leaves of *Arabidopsis* [38], which may due to their specialized function in plant development. Thus, it could be hypothesized that Cl*RLT1* might have other functions apart from branching regulation in the development of Chinese Fir.

RNase IIIs play important roles in maturation of almost every class of RNA, post-transcriptional gene expression control [39,40,41], cellular defense against viral infection [42] and RNA interference (RNAi) [43,44]. *Arabidopsis* RTL2 recognizes and cleaves dsRNAs, yielding small RNAs ~25 nt or longer [45]. Dicer is a well-known RNase III that processes dsRNAs to siRNAs ~20 bp long that act as effectors during RNAi [46]. *DCL1*, *DCL2*, *DCL3*, and *DCL4* of *Arabidopsis* play roles in siRNA synthesis [47,48]. *E. coli* RNase III can affect gene expression as a processing enzyme or as a binding protein [23,49,50,51,52,53,54,55]. We hypothesize that ClRTL1 affects shoot branching in Chinese fir by regulating the expression of other branching-related genes.

### 3.3. ClRTL1 Encodes a mini-RNase III Protein with Conserved RNase III Enzyme Activity But with a Different Mechanism of RNA Recognition from the Typical RNase III

Cl*RTL1* was identified as a RNase III-encoding gene based on its conserved nine-amino-acid, RNase III signature motif ATLAYLGDC (counterpart to Aa-RNase III ETLEFLGDA) of deduced amino-acid sequences. The simplest RNase III usually contains two important domains, an N-terminal endonuclease domain (endoND) and a double-stranded RNA binding domain (dsRBD) [20,56]. However, the second structure and alignment of the deduced amino acid sequence of ClRTL1 with *A. aeolicus* RNase III, *T. maritima* RNase III, *B. cereus* mini-RNase III and *B. subtilis* mini-RNase III showed that the ClRTL1 contained only the endonuclease domain without the dsRNA binding domain and the linker domain between them. ClRTL1 also resembled *B. cereus* mini-RNase III more than *A. aeolicus* RNase III, so Cl*RTL1* is classified as *C. lanceolata* mini-RNase III-like protein. Residues equivalent to the *A. aeolicus* RNase III D44 and E110 are essential to the dsRNA processing center responsible for the dsRNA cleavage activity, which are strictly conserved in all family members [28]. When the conserved amino acid D44 and E110 in the RNase III motif mutated, the protein lost its RNase III activity [57,58]. Clearly, D44 and E110 in ClRTL1 are conserved. Therefore, we deduced that these amino acids formed the catalytic center and conferred on ClRTL1 the RNase III activity. This finding agreed with the relationship displayed in the phylogenic tree, which indicates that the RNase III protein was probably conserved in the analyzed organisms (Figure 6). Moreover, according to the phylogenetic tree, ClRTL1 was clustered into the group of mini-RNase III, which implied its function in plant development as a mini-RNase III protein.

However, the second structure analysis, especially the absence of dsRBD and the linker domain implied that ClRTL1 had a different mechanism of RNA recognition from typical RNase IIIs. dsRBD plays the most important role in the binding of dsRNA. The linker (145EGRVKKD 151 of *A. aeolicus*) between the endoND and dsRBD is flexible, which allows dsRBD to rotate and shift, enabling RNase III to assume at least two distinct dsRNA binding modes [28]. However, many RNase III molecules without dsRBD can still function with a different mechanism of dsRNA recognition. *B. subtilis* mini RNase III, which lacks a dsRBD, still cleaves dsRNA [25]. A study by the Nicholson laboratory showed that truncated *E. coli* RNase III, which lacks dsRBD, could accurately cleave dsRNA at low salt concentrations and also retain strict specificity for dsRNA [57]. *A. aeolicus* RNase III monomer contacts with dsRNA in four places. Two RNA binding motifs (RBM1 and RMB2) are in dsRBD, and two (RBM3 and RMB4) are in the catalytic domain [23]. However, *B. cereus* mini-RNase III only has one (RBM3) of the four RNA binding motifs. Thus, a model of dsRNA recognition by *B. cereus* mini-RNase III was proposed; in this model, the loops between α5b and α6 was ideally placed to make contacts with the major groove over the single turn in the RNA helix with the key amino acid residues K86 and N87. A typical RNase III makes primarily minor groove contacts (with the exception of RBM3), spreading over two complete helical turns of RNA [25]. According to our analysis, ClRTL1 also had only one (RBM3) of the four RNA binding motifs with an extra α5b helix instead of RBM4 at the same station. In addition, the key amino acid Lys and Asn (K86 and N87, *A. aeolicus* RNase III numbering) were conserved in the loops between α5b and α6 in ClRNase III-like protein. Thus, we proposed that ClRTL1 shared a similar dsRNA recognition with *B. subtilis* mini-RNase III and *E. coli* truncated RNase III.

In addition, Mg^2+^ is essential for the cleavage activity of RNase III; however, E40 and D107 (*A. aeolicus* numbering), which played a role in Mg^2+^ binding to RNase III, are absent from ClRNase III-like protein as in *B. subtilis* mini-RNase III protein [29,30,31,32]. Thus, alternative means of binding metal ion may exist in Cl RNase III like protein that is different from typical RNase III. Taken together, the different mechanism of dsRNA recognition raised the possibility that ClRTL1 may function differently from other typical RNase IIIs and may confer its specificity for regulating certain genes.

## 4. Experimental Section

### 4.1. Plant Materials

Two-year-old cuttings of Chinese fir elite genotype No. 020 (wild type) and mutant “Dugansha” were grown in the nursery fields of Nanjing Forestry University in Jiangsu Province, Eastern China (Figure 1C,D). Needles, stems, roots, secondary SAMs, male and female cones, primary SAMs from 15 April 2009 and 6 May 2009 of Chinese fir elite genotype No. 020, and primary SAMs from 6 May of the mutant “Dugansha” (no secondary buds developed for mutant) were all harvested and stored at −80 °C until use. *Arabidopsis* wild-type and transgenic plants were cultivated in a growth room at 22 °C with a 16 h light/8 h dark photoperiod.

### 4.2. Screening for Branching-Related Genes with the cDNA-AFLP System

Total RNA was extracted using the cetyl trimethyl ammonium bromide (CTAB)–LiCl method [59]. Double-stranded cDNA was synthesized with the Takara superscript reverse transcriptase combined with the replacement synthesis method. The cDNA fragments related to shoot branching in Chinese fir were isolated using the optimized cDNA–AFLP technique as described by Bachem *et al.* [26,60]. A total of 300 ng of cDNA was digested with the restriction enzymes Mse I and EcoR I and then ligated to adaptors (Table 1). For pre-amplification, Mse I primers without selective nucleotides combined with EcoR I primers without selective nucleotides were used. The obtained amplification mixtures were diluted 20-fold, and 2 µL was used for selective amplification. All possible primer combinations with two selective nucleotides were used for transcript profiling (Table 1). The amplified products were separated on 6% polyacrylamide gels running at 85 W for about 2 h. Gels were silver stained according to the standard protocol [60], and the images were captured with scanning. Bands corresponding to 200–1000 bp-long differentially expressed transcripts, which were identified as potential shoot branching-related genes, were isolated from the gels. The eluted DNA was reamplified under the same conditions as that for the selective amplification. Reamplified products representing the branching-related, transcript-derived fragments (TDFs) were verified on a 2% agarose gel and directly sequenced with the selective Mse I primer.

**Table 1:** Adapters and primers used in this study.

Primer/Adapter	Nucleotide Sequences
*EcoR I Adapters*	5′-CTCGTAGACTGCGTACC-3′
	3′-CTGACGCATGGTTAA-5′
*Mse I Adapters*	5′-GACGATGAGTCCTGAG-3′
	3′-TACTCAGGACTCAT-5′
*Pre-amplification primer E 00*	5′-GACTGCGTACCAATTC-3′
*Pre-amplification primer M 00*	5′-GATGAGTCCTGAGTAA-3′
*Selective-amplification primers E + 2*	5′-GACTGCGTACCAATTCNN-3′
*Selective-amplification primers M + 2*	5′-GATGA GTCCTGAGTAANN-3′
*GSP1*	5′-TGATGCCCTGTTATACACTGCTGCTCC-3′
*GSP2*	5′-TGGAGCAGCAGTGTATAACAGGGCATCATCT-3′
*RNase III F*	5′-GCGAACGAGGATGGCTGCTAC-3′
*RNase III R*	5′-ATCCCATCCTAACACCACTTG-3′
*RNase III forward primer R1*	5′-GAACAGTAAGGCGTGCTGGAG-3′
*RNase III reverse primer R2*	5′-CAAAACCCGAGGCTTCTCATT-3′
*Actin forward primer A1*	5′-CAGCAACTGGGATGATATGG-3′
*Actin reverse primer A2*	5′-ATTTCGCTTTCAGCAGTGGT-3′
*RNase III forward primer F4*	5′-CTCCCATGACCAACCTGTTTCTG-3′
*RNase III forward primer R4*	5′-ATTAAGGCATCCAAGCGATTTCC-3′

### 4.3. Cloning and Sequence Analysis of the Full-Length ClRTL1 cDNA Sequence

Using the BLAST network service, one of the gene fragments was found to be similar to *A. thaliana* RNase III gene with an *E*-value of 2 × 10 ^−61^. The 5′- and 3′-ends of the cDNA sequence were obtained using SMARTer™ RACE cDNA Amplification Kit (Clontech, Beijing, China) according to the manufacturer’s instructions. Double-stranded cDNA was synthesized from 1 μg of RNA. Oligonucleotide primers GSP1 and GSP2 were designed to amplify the Cl*RTL1* 5′- and 3′-cDNA ends (Table 1). The products of 5′-RACE and 3′-RACE products were subcloned into the pMD™^19-T^ vector (Takara, Dalian, China) and sequenced. The full-length cDNA sequence was amplified with end-to-end PCR using primers RNase III F and RNase III R (Table 1). BLASTN, PSI-BLAST, and BLASTP (together with the PDB data bank) were used to evaluate nucleotide identity and amino-acid sequence similarity of Cl*RTL1*. Protein domain analysis was performed with the Simple Modular Architecture Research Tool [61].

### 4.4. Secondary Structural Analysis of the Deduced Protein ClRTL1

The secondary structure of the deduced protein Cl*RTL1* was analyzed using tools available at the Institut de Biologie et Chimie des Protéines Network Protein Sequence Analysis website [62]. The secondary structures of *A. aeolicus* RNase III ortholog (PDB: 2NUE), *T. maritima* RNase III ortholog (PDB: 10OW), and *B. cereus* mini-RNase III (PROTEIN DATA BANK 1U61) were illustrated using Swiss-Pdbviewer [63].

### 4.5. Relative Expression Analysis of ClRTL1

To further verify the isolation efficiency with cDNA-AFLP and the function of Cl*RTL1* in Chinese fir shoot branching, we performed gene expression analysis in Chinese fir No. 020 primary SAMs that had not yet initiated AMs, in Chinese fir No. 020 primary SAMs that were initiating AMs, and in the mutant “Dugansha” primary SAMs using semi-quantitative reverse-transcription PCR. Total RNA was extracted using CTAB-LiCl method [59]. cDNA was synthesized using the Takara superscript reverse transcriptase combined with replacement-synthesis method. The primers R1 and R2 are shown in Table 1.

To analyze the expression of different tissues, real-time PCR analysis was carried out using the gene-specific primers R1 and R2 (Table 1). Total RNA was isolated separately from needles, primary stems (1 cm below the primary SAM), secondary stems (7 cm below the primary SAM), roots, secondary SAMs, and male and female cones of Chinese fir elite genotype No. 020 using CTAB-LiCl method [59]. Double-stranded cDNA was synthesized with PrimeScript RT Reagent Kit (Takara, Dalian, China). Real-time PCR analysis was conducted using Power SYBR Green PCR Master Mix (Applied Biosystems Foster City, CA, USA). Each reaction contained 50 ng of cDNA, 1 μL of 10 μM gene-specific primers, and 25 μL of Master mix. Amplification was performed in a Light Cycler Instrument (Bio-Rad iQ5, Hercules, CA, USA). Actin was amplified as an internal standard with primers A1 and A2 (Table 1).

### 4.6. Construction of Expression Vectors and Plant Transformation into A. thaliana

The full-length Cl*RTL1* cDNA was cloned into the pMD19-T vector and sequenced. After digestion of the pMD19-T^ClRNase III^ vectors with *Sal* I and *Sac* I, the fragments were subcloned into pCAMBIA1301 binary vector under the control of cauliflower mosaic virus (CaMV) 35S promoter. Subsequently, the pCAMBIA1301^Cl*RTL1*^ plasmid was mobilized into competent cells of *Arobacterium tumefaciens* EHA105 using freeze–thaw method [64]. Finally, the constructs were introduced into *A. thaliana* using floral-dip method [65].

### 4.7. Isolation of 35S: Cl RNase III Transgenic Arabidopsis Lines

Transgenic lines were selected on half-strength MS medium [66] containing 50 mg/L hygromycin B. PCR and reverse-transcription PCR were carried out to confirm the integration of Cl*RTL1* into *A. thaliana* chromosomal DNA. Total genomic DNA was isolated from hygromycin-resistant plants using CTAB method. Total RNA was extracted using CTAB–LiCl method [59]. cDNA was synthesized with the Takara superscript reverse transcriptase combined with replacement-synthesis method. The primers used to amplify a 500 bp fragment to detect the presence of Cl*RTL1* were F4 and R4 (Table 1). T_2_ plants exhibited 3:1 segregation of kanamycin resistance marker, indicating the presence of only one insertion in each T_1_ transgenic line. To identify heterozygotes and homozygotes of each line, T_2_ plants were grown until seeds were obtained. Then, segregation analysis of kanamycin resistance marker in T_3_ generation plants was conducted. T_2_ plants that showed 3:1 segregation of kanamycin resistance marker were identified as heterozygotes. T_2_ plants that showed no segregation were identified as homozygotes [67].

## 5. Conclusions

We isolated and characterized Cl*RTL1* from Chinese fir and suggested that it was involved in plant shoot branching. We showed that the gene encoded a mini-RNase III like protein with conserved RNase III activity but a different mechanism of RNA recognition model from typical RNase III proteins. Overexpression of this gene in *Arabidopsis* enhanced shoot branching both in rosette branches and cauline branches. The mechanism of Cl*RTL1* promoting shoot branch might be increasing axillary bud outgrowth by affecting the expression of other branching-related genes. However, only preliminary function analysis was performed in this study. Therefore, further investigation on the function of this novel gene and its interaction with other branching-related genes is warranted.

## Supplementary Materials

Supplementary materials can be found at http://www.mdpi.com/1422-0067/16/10/25691/s1.

## References

[1] McSteen P., Leyser O. (2005). Shoot branching. Annu. Rev. Plant Biol..

[2] Mason M.G., Ross J.J., Babst B.A., Wienclaw B.N., Beveridge C.A. (2014). Sugar demand, not auxin, is the initial regulator of apical dominance. Proc. Natl. Acad. Sci. USA.

[3] Yaish M.W.F., Guevara D.R., El-Kereamy A., Rothstein S.J., Pua E.C., Davey M.R. (2010). Axillary Shoot Branching in Plants. Plant Developmental Biology-Biotechnological Perspectives.

[4] Schumacher K., Schmitt T., Rossberg M., Schmitz G., Theres K. (1999). The Lateral suppressor (Ls) gene of tomato encodes a new member of the VHIID protein family. Proc. Natl. Acad. Sci. USA.

[5] Greb T., Clarenz O., Schafer E., Muller D., Herrero R., Schmitz G., Theres K. (2003). Molecular analysis of the LATERAL SUPPRESSOR gene in *Arabidopsis* reveals a conserved control mechanism for axillary meristem formation. Genes Dev..

[6] Li X., Qian Q., Fu Z., Wang Y., Xiong G., Zeng D., Wang X., Liu X., Teng S., Hiroshi F. (2003). Control of tillering in rice. Nature.

[7] Schmitz G., Tillmann E., Carriero F., Fiore C., Cellini F., Theres K. (2002). The tomato Blind gene encodes a MYB transcription factor that controls the formation of lateral meristems. Proc. Natl. Acad. Sci. USA.

[8] Keller T., Abbott J., Moritz T., Doner P. (2006). *Arabidopsis* REGULATOR OF AXILLARYMERISTEMS1 controls a leaf axil stem cell niche and modulates vegetative development. Plant Cell.

[9] Komatsu K., Maekawa M., Ujiie S., Satake Y., Furutani I., Okamoto H., Shimamoto K., Kyozuka J. (2003). LAX and SPA: Major regulators of shoot branching in rice. Proc. Natl. Acad. Sci. USA.

[10] Hubbard L., McSteen P., Doebley J., Hake S. (2002). Expression patterns and mutant phenotype of teosinte brancbedl correlate with growth suppression in maize and teosinte. Genetics.

[11] Takeda T., Suwa Y., Suzukj M., Kitano H., Ueguchi-Tanaka M., Ashikari M., Matsuoka M. (2003). The OsTB1 gene negatively regulates lateral branching in rice. Plant.

[12] Braun N., de Saint Germain A., Pillot J.-P., Boutet-Mercey S., Dalmais M., Antoniadi I., Li X., Maia-Grondard A., le Signor C., Bouteiller N. (2012). The pea TCP transcription factor PsBRC1 acts downstream of strigolactones to control shoot branching. Plant Physiol..

[13] Reintanz B., Lehnen M., Reichelt M., Gershenzon J., Kowalczyk M., Sandberg G., Godde M., Uhl R., Palme K. (2001). bus, a Bushy *Arabidopsis* knockout mutant with abolished synthesis of short-chain aliphatic glucosinolates. Plant Cell.

[14] Tantikanjana T., Young J.W.H., Letham D.S., Griffith M., Hussain M., Ljung K., Sandberg G., Sundaresan V. (2001). Control of axillary bud initiation and shoot architecture in *Arabidopsis* through the SUPERSHOOT gene. Genes Dev..

[15] Nakagawa H., Jiang C.J., Sakakibara H., Kojima M., Honda I., Ajisaka H., Nishijima T., Koshioka M., Homma T., Mander L.N. (2005). Overexpression of a petunia zinc-finger gene alters cytokinin metabolism and plant forms. Plant J..

[16] Gong L., Yang Y.J., Zhou J. (2012). Genes involved in the synthesis and signaling pathway of strigolactone, a shoot branching inhibitor. Biol. Plant..

[17] Chen Y.W., Shi J.S. (1983). Some fundamental strategies on Chinese fir genetic improvement (I). J. Nanjing For. Univ..

[18] Filippov V., Solovyev V., Filippva M., Gill S.S. (2000). A novel type of RNase III family proteins in eukaryotes. Gene.

[19] Robertson H.D., Webster R.E., Zinder N.D. (1968). Purification and properties of ribonuclease III from *Escherichia coli*. J. Biol. Chem..

[20] Elela S.A., Igel H., Ares M. (1996). RNase III cleaves eukaryotic preribosomal RNA at a U3 snoRNP-dependent site. Cell.

[21] Jacobsen S.E., Running M.P., Meyerowitz E.M. (1999). Disruption of an RNA helicase/RNAse III gene in *Arabidopsis* causes unregulated cell division in floral meristems. Development.

[22] Wu H., Xu H., Miraglia L.J., Crooke S.T. (2000). Human RNase III is a 160 kDa protein involved in preribosomal RNA processing. J. Biol. Chem..

[23] Gan J., Tropea J.E., Austin B.P., Court D.L., Waugh D.S., Ji X. (2006). Structural insight into the mechanism of double-stranded RNA processing by ribonuclease III. Cell.

[24] Bernstein E., Caudy A.A., Hammond S.M., Hannon G.J. (2001). Role for a bidentate ribonuclease in the initiation step of RNA interference. Nature.

[25] Redko Y., Bechhofer D.H., Condon C. (2008). Mini-III, an unusual member of the RNase III family of enzymes, catalyses 23S ribosomal RNA maturation in *B. subtilis*. Mol. Microbiol..

[26] Li X., Shi J.S. Development and Optimization of cDNA-AFLP Reaction System for Young Stem of *Cunninghamia lanceolata* (Lamb.) Hook. Proceedings of the International Conference on Plant Vascular Biology and Agriculture.

[27] Ray A., Lang J.D., Golden T., Ray S. (1996). SHORT INTEGUMENT (SIN1), a gene required for ovule development in *Arabidopsis*, also controls flowering time. Development.

[28] Blaszczyk J., Tropea J.E., Bubunenko M., Routzahn K.M., Waugh D.S., Court D.L., Ji X. (2001). Crystallographic and modeling studies of RNase III suggest a mechanism for double-stranded RNA cleavage. Structure.

[29] Li H.L., Chelladurai B.S., Zhang K., Nicholson A.W. (1993). Ribonuclease III cleavage of a bacteriophage T7 processing signal. Divalent cation specificity, and specific anion effects. Nucleic Acids Res..

[30] Rotondo G., Frendewey D. (1996). Purification and characterization of the Pac1 ribonuclease of Schizosaccharomyces pombe. Nucleic Acids Res..

[31] Provost P., Dishart D., Doucet J., Frendewey D., Samuelsson B., Rådmark O. (2002). Ribonuclease activity and RNA binding of recombinant human Dicer. EMBO J..

[32] Kreuze J.F., Savenkov E.I., Cuellar W., Li X., Valkonen J.P. (2005). Viral class 1 RNase III involved in suppression of RNA silencing. J. Virol..

[33] Dellagi A., Birch P.R.J., Heilbronn J., Lyon G.D. (2000). cDNA-AFLP analysis of differential gene-expression in the prokaryotic plant pathogen Erwinia-Carotovora. Microbiology.

[34] Durrant W.E., Rowland O., Piedras P., Hammondkosack K.E., Jones J.D.G. (2000). cDNA-AFLP reveals a striking overlap in race-specific resistance and wound response gene-expression profiles. Plant Cell.

[35] Van der Biezen E.A., Juwana H., Parker J.E., Jones J.D.G. (2000). cDNA-AFLP display for the isolation of Peronospora parasitica genes expressed during infection in *Arabidopsis thaliana*. Mol. Plant Microbe Interact..

[36] Golden T.A., Schauer S.E., Lang J.D., Pien S., Mushegian A.R. (2002). Short integuments1/suspensor1/carpel factory, a Dicer homolog, is a maternal effect gene required for embryo development in *Arabidopsis*. Plant Physiol..

[37] Schauer S.E., Jacobsen S.E., Meinke D.W., Ray A. (2002). DICER-LIKE1: Blind men and elephants in *Arabidopsis* development. Trends Plant Sci..

[38] Xie Z., Allen E., Wilken A., Carrington J.C. (2005). DICER-LIKE 4 functions in trans-acting small interfering RNA biogenesis and vegetative phase change in *Arabidopsis thaliana*. Proc. Natl. Acad. Sci. USA.

[39] Court D., Belasco J., Brawerman G. (1993). RNA processing and degradation by RNase III. Control of Messenger RNA Stability.

[40] Nicholson A.W., Hannon G.J. (2003). The ribonuclease III family: forms and functions in RNA maturation, decay, and gene silencing. RNAi: A Guide to Gene Silencing.

[41] Lee Y., Ahn C., Han J.J., Choi H., Kim J., Yim J., Lee J., Provost P., Radmark O., Kim S. (2003). The nuclear RNase III Drosha initialtes micro RNA processing. Nature.

[42] Lichner Z., Silhavy D., Burgya’n J. (2003). Double-stranded RNA binding proteins could suppress RNA interference-mediated antiviral defences. J. Gen. Virol..

[43] Carthew R.W. (2001). Gene silencing by double-stranded RNA. Curr. Opin. Cell Biol..

[44] Carmell M.A., Hannon G.J. (2004). RNase III enzymes and the initiation of gene silencing. Nat. Struct. Mol. Biol..

[45] Kiyota E., Okada R., Kondo N., Hiraguri A., Moriyama H., Fukuhara T. (2011). An *Arabidopsis* RNase III-like protein, AtRTL2, cleaves double-stranded RNA *in vitro*. J. Plant Res..

[46] Zhang H., Kolb F.A., Jaskiewicz L., Westhof E., Filipowicz W. (2004). Single processing center models for human Dicer and bacterial RNase III. Cell.

[47] Dunoyer P., Himber C., Voinnet O. (2005). DICER-LIKE 4 is required for RNA interference and produces the 21-nucleotide small interfering RNA component of the plant cell-to-cell silencing signal. Nat. Genet..

[48] Mlotshwa S., Pruss G.J., Peragine A., Endres M.W., Li J., Chen X., Scott Poethig R., Bowman L.H., Vance V. (2008). DICER-LIKE2 plays a primary role in transitive silencing of transgenes in *Arabidopsis*. PLoS ONE.

[49] Dunn J.J., Boyer P.D. (1982). Ribonuclease III. The Enzymes.

[50] Robertson H.D. (1982). *Escherichia coli* ribonuclease III cleavage sites. Cell.

[51] Robertson H.D., Dunn J.J. (1975). Ribonnucleic acid processing activity of *Escherichia coli* ribonuclease III. J. Biol. Chem..

[52] Calin-Jageman I., Nicholson A.W. (2003). RNA structure dependent uncoupling of substrate recognition and cleavage by *Escherichia coli* ribonuclease III. Nucleic Acids Res..

[53] Dasgupta S., Fernandez L., Kameyama L., Inada T., Nakamura Y., Pappas A., Court D.L. (1998). Genetic uncoupling of the dsRNA-binding and RNA cleavage activity of the *Escherichia coli* endoribonuclease RNase III-the effect of dsRNA binding on gene expression. Mol. Microbiol..

[54] Guarneros G. (1988). Retroregulation of bacteriophage lambda int gene expression. Curr. Top. Microbiol. Immunol..

[55] Oppenheim A.B., Komitzer D., Altuvia S., Court D.L. (1993). Posttranscriptional control of the lysogenic pathway in bacteriophage lambda. Prog. Nucleic Acid Res. Mol. Biol..

[56] Kharratt A., Macia M.J., Gibson T.J., Nilges M., Pastore A. (1995). Structure of the dsRNA binding domain of *E. coli* RNase III. EMBO J..

[57] Sun W., Jun E., Nicholson A.W. (2001). Intrinsic double-stranded-RNA processing activity of *Escherichia coli* ribonuclease III lacking the dsRNA-binding domain. Biochemistry.

[58] Sun W., Jun E., Nicholson A.W. (2004). Mutational analysis of the nuclease domain of *Escherichia coli* ribonuclease III. Identification of conserved acidic residues that are important for catalytic function *in vitro*. Biochemistry.

[59] Chang S.J., Jeff P., John C. (1993). A simple and efficient method for isolating RNA from pine trees. Plant Mol. Biol. Rep..

[60] Bachem C.W.B., van der Hoeven R.S., de Bruijn S.M., Vreugdenhil D., Zabeau M., Visser R.G.F. (1996). Visualization of differential gene expression using a novel method of RNA fingerprinting based on AFLP: Analysis of gene expression during potato tuber development. Plant J..

[61] SMART. http://smart.embl.de/.

[62] Combet C., Blanchet C., Geourjon C., Deleage G. (2000). NPS@: Network protein sequence analysis. Trends Biochem. Sci..

[63] ExPASy. http://expasy.org/spdbv/.

[64] Hoekema A., Hirsch P.R., Hooykaas P.J.J., Schilperoort R.A. (1983). A binary plant vector strategy based on separation of vir- and T-region of the *Agrobacterium tumefaciens* Ti-plasmid. Nature.

[65] Bechtold N., Pelletier G. (1998). In planta Agrobacterium-mediated transformation of adult *Arabidopsis thaliana* plants by vacuum infiltration. Methods Mol. Biol..

[66] Murashige T., Skoog F. (1962). A revised medium for rapid growth and bioassays with tobacco tissue cultures. Physiol. Plant.

[67] Sridevi G., Parameswari C., Rajamuni P., Veluthambi K. (2006). Identification of hemizygous and homozygous transgenic rice plants in T1 generation by DNA blot analysis. Plant Biotechnol..

