# Supplementary material for: ClRTL1 Encodes a Chinese Fir RNase III–Like Protein Involved in Regulating Shoot Branching
Source: Int J Mol Sci. 2015 Oct 26;16(10):25691–710. doi: 10.3390/ijms161025691 (PMC4632822; doi:10.3390/ijms161025691)
Supplement: Supplementary file 1 [file ijms-16-25691-s001.pdf]

Supplementary Information

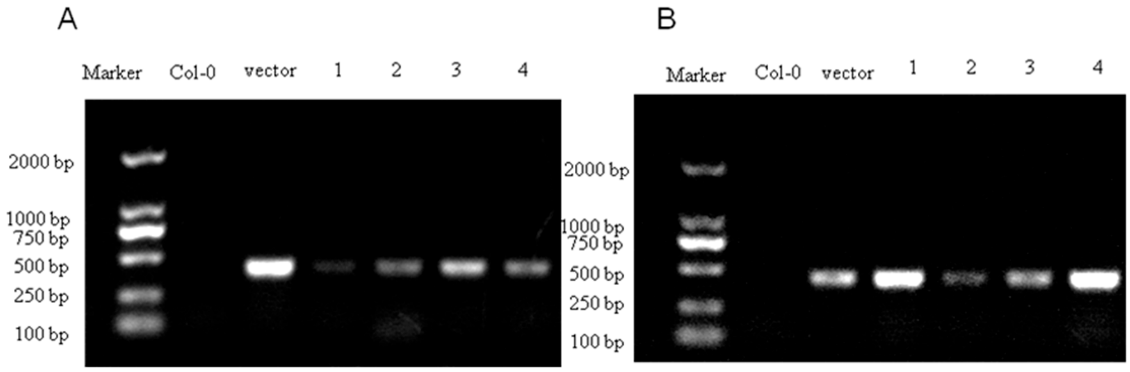

Figure S1. Confirmation of the integration of Cl*RTL1* into *Arabidopsis thaliana* by PCR and RT-PCR. (**A**) PCR analysis of 35S: Cl*RTL1* plants. Col-0: wild-type *Arabidopsis thaliana* (ecotype Columbia); vector: the plant expression vector CaMV35S-PBI121 Cl*RTL1*; lanes 1–4: transformant T1 plants; (**B**) RT-PCR analysis of 35S: Cl*RTL1* plants. Col-0: wild-type *Arabidopsis thaliana* (ecotype Columbia); vector: the plant expression vector CaMV35S-PBI121 Cl*RTL1*; lanes 1–4: transformant T1 plants.

Table S1. Numbers of rosette leaves of Col-0 and 35S: Cl*RTL1 Arabidopsis*.

**Types of *Arabidopsis Thaliana***	**Col-0**	**Heterozygous 35S: Cl*RTL1***	**Homozygous 35S: Cl*RTL1***
Number of rosette leaves	7 ± 3	7 ± 6.1	10 ± 3.5
